# A Novel Polysaccharide in Insects Activates the Innate Immune System in Mouse Macrophage RAW264 Cells

**DOI:** 10.1371/journal.pone.0114823

**Published:** 2014-12-09

**Authors:** Takashi Ohta, Atsushi Ido, Kie Kusano, Chiemi Miura, Takeshi Miura

**Affiliations:** Research Group for Reproductive Physiology, Southern Ehime Fisheries Research Center, Ehime University, Ainan, Ehime, Japan; Institute of Microbial Technology, India

## Abstract

A novel water-soluble polysaccharide was identified in the pupae of the melon fly (*Bactrocera cucurbitae*) as a molecule that activates the mammalian innate immune response. We attempted to purify this innate immune activator using nitric oxide (NO) production in mouse RAW264 macrophages as an indicator of immunostimulatory activity. A novel acidic polysaccharide was identified, which we named “dipterose”, with a molecular weight of 1.01×10^6^ and comprising nine monosaccharides. Dipterose was synthesized in the melon fly itself at the pupal stage. The NO-producing activity of dipterose was approximately equal to that of lipopolysaccharide, a potent immunostimulator. Inhibition of Toll-like receptor 4 (TLR4) led to the suppression of NO production by dipterose. Furthermore, dipterose induced the expression of proinflammatory cytokines and interferon β (IFNβ) and promoted the activation of nuclear factor kappa B (NF-κB) in macrophages, indicating that it stimulates the induction of various cytokines in RAW264 cells via the TLR4 signaling pathway. Our results thus suggest that dipterose activates the innate immune response against various pathogenic microorganisms and viral infections. This is the first identification of an innate immune-activating polysaccharide from an animal.

## Introduction

Insects have great potential as a source of food and feed [1] and a nutrition source that is comparable to meat and fish [2]. Insects could become a future main source of animal protein for stock farming, poultry farming, and fish culture (e.g., fish meal) [3]. As the technology required for the large-scale production of several insect species such as the silkworm and bee has already been established, the contribution of insects to global food and feed supply problems is anticipated [1]. It is noteworthy in this regard that various bioactive substances are found in numerous insect species with beneficial characteristics [4]–[7]. These substances include compounds with diverse pharmacological properties, including anti-cancer, anti-tumor, anti-viral, and anti-microbial activities and the capacity to enhance resistance to various diseases in humans, livestock, poultry, and fish [8]–[12].

Exogenous glycans can activate the innate immune response against various pathogens [13]. Species-specific monosaccharide variations and glycan structures are recognized through germline-encoded pattern recognition receptors (PRRs), such as Toll-like receptors (TLRs), that directly activate innate immune cells [14]–[19]. Previous studies have reported that various glycans extracted from natural plants and fungi pharmacologically function as immunostimulators because their exoskeleton is formed by cell walls composed of polysaccharides [20], [21]. Chitin and chitin derivatives, which are polymerized by N-acetyl-β-D-glucosamine and form the exoskeletons of arthropods such as insects, activate the innate immune response in mammals [22]. However, no other insect glycans have shown immunostimulatory activity.

In our current study, we describe the identification of a novel water-soluble polysaccharide from Diptera melon fly pupae that activates the innate immune system in mouse macrophage RAW264 cells. We have named this polysaccharide “dipterose”. Surprisingly, dipterose is predominately composed of L-rhamnose, despite the inability of animals, including insects, to synthesize this saccharide. We demonstrate the mechanism of activation of the innate immune system by dipterose.

## Materials and Methods

### Preparation of melon fly pupal extract

Melon fly (*Bactrocera cucurbitae*) pupae were provided by the Okinawa Prefectural Plant Protection Center (Okinawa, Japan). These pupae were autoclaved for 20 min at 121°C to inactivate endogenous enzymes and then frozen at –30°C until use. The frozen pupae (2.6 kg) were homogenized using a mixer for 5 min. The resulting homogenate was diluted with four volumes of ultrapure water and mixed gently for 1 h at room temperature. The supernatant obtained by centrifugation at 20,000×g for 1 h was concentrated to a 500 mL volume by evaporation on a water bath at 50–60°C.

### Purification of polysaccharides from melon fly pupae

Melon fly pupal extracts were added to four volumes of 100% (v/v) methanol and gently mixed overnight at 4°C to precipitate the polysaccharides. The precipitate was collected by centrifugation, washed successively three times with 100% methanol, and dried under decompression. The precipitate was then added to 500 mL of 20 mM Tris-HCl (pH 8.0) and mixed overnight at 4°C to dissolve the polysaccharides. The precipitate was then removed to obtain a crude polysaccharide preparation which was applied to a DEAE-cellulose (DE52) anion-exchange chromatography column (2.5 cm×10 cm) (Whatman, Kent, UK) pre-equilibrated with 20 mM Tris-HCl (pH 8.0). Fractions were prepared via sequential elution with increasing concentrations of NaCl (0.2, 0.5, and 1.0 M) solution at a flow rate of 2.0 mL/min. The eluted 10-mL fractions were collected and assayed for NO-producing activity in RAW264 cells and for total carbohydrates using the phenol-sulfuric acid method with a glucose standard [23]. Fractions containing NO-producing activity were pooled and precipitated using four volumes of 100% (v/v) methanol overnight at 4°C. The precipitate was then separated by centrifugation and dissolved in 20 mM Tris-HCl (pH 8.0) containing 500 mM NaCl.

The polysaccharides separated using anion-exchange chromatography were fractionated by gel filtration chromatography on a Superose 6 column (10/300; GE Healthcare UK Ltd., Buckinghamshire, UK). The column was equilibrated with 20 mM Tris-HCl (pH 8.0) containing 500 mM NaCl and eluted with the same solution. The flow rate through the column was 0.5 mL/min and 0.5-mL fractions were collected at regular intervals. The eluted fractions were assayed as detailed above. The active fractions were pooled and precipitated by the addition of four volumes of 100% (v/v) methanol overnight at 4°C. The resulting precipitate obtained by centrifugation was lyophilized to obtain bioactive polysaccharide.

### Determination of polysaccharide molecular weights

Polysaccharide molecular weights were determined by gel filtration chromatography using a high-performance liquid chromatography instrument (Hitachi, Tokyo, Japan). The isolated melon fly polysaccharide (500 µg) was dissolved in 500 µL of 0.2 M phosphate buffer (PB) (pH 7.5) and filtered with a 0.22 µm filter applied to a Shodex SB-807 HQ size-exclusion chromatography column (Showa Denko K.K., Tokyo, Japan), maintained at a temperature of 35°C. It was then eluted from the column with 0.2 M PB (pH 7.5) at a flow rate of 0.5 mL/min and detected by a refractive index detector. Preliminary calibration of the column was conducted using pullulans of different molecular weights (pullulan P-5, P10, P-20, P50, P100, P200, P-400, P-800, and P-2500). The molecular weight of the polysaccharide was calculated from the calibration curve generated using these standard pullulans.

### Monosaccharide composition of the melon fly bioactive polysaccharide

Determination of the monosaccharide composition of the melon fly bioactive polysaccharide was performed in accordance with the method of Akiyama *et al*. [24]. Briefly, polysaccharide (500 µg) was hydrolyzed with 2 M trifluoroacetic acid (TFA) at 100°C for 16 h. The hydrolyzed products were then evaporated using N_2_ stream and the residue was added to a mixture of ethanethiol and TFA (2∶1, 100 µL) and incubated at room temperature for 10 min. After incubation, trimethylsilylation pyridine (250 µL) was added, followed by hexamethyldisilazane (500 µL) and TFA (150 µL). The mixtures were kept for 1 h at room temperature. The reaction mixtures were evaporated using N_2_ stream. After the dried products were mixed with deionized water (250 µL), hexane (500 µL) was added for extraction. Approximately 1 µL of clear supernatant was then injected into a gas chromatography column. Gas chromatography-mass spectrometry (GC-MS) analyses were performed on an Agilent 7890A gas chromatograph (Agilent Technologies, Santa Clara, CA) equipped with a HP-5 capillary column (30 m×0.35 mm×0.25 µm) connected to a JEOL MS-1050Q instrument (JEOL, Tokyo, Japan). Helium was used as the carrier gas at a constant flow of 1 mL/min. The injector temperature was kept constant at 250°C and the column temperature was programmed as follows: oven start temperature of 165°C, which was gradually increased to 235°C (2°C/min) and then 300°C (10°C/min, then held for 10 min). The mass spectrometry acquisition parameters included scanning from *m/z* 40 to 600 in the electron impact mode for routine analysis.

### Cell culture

RAW264 cells (a murine macrophage line) were obtained from the Cell Bank RIKEN BioResource Center (Tsukuba, Japan). Cells were grown in MEM supplemented with 10% fetal bovine serum, 0.1 mM non-essential amino acids, 100 U/mL penicillin, and 100 µg/mL streptomycin. Cultures were maintained at 37°C in a 5% CO_2_ humidified atmosphere.

### NO assay

The levels of NO in the macrophage culture medium were measured using a Griess reagent system kit (Promega, Madison, WI) in accordance with the manufacturer’s instructions. Cells were plated at 10^6^ cells/mL in 96-well plates and stimulated with lipopolysaccharide (LPS) or melon fly pupal extract for 20 h. Fifty microliter aliquots of culture medium supernatant were then gently mixed with an equal volume of sulfanilamide solution and incubated in the dark at room temperature for 10 min. After this incubation, 50 µL of NBT solution was added and the reaction solution was incubated in the dark at room temperature for a further 10 min. The absorbance of this solution at 540 nm was then measured in a microplate reader and the nitrite concentration was calculated from a NaNO_2_ standard curve.

### TLR2 and TLR4 blocking experiments

RAW264 cells were plated as described above and then preincubated for 1 h at 37°C (5% CO_2_) with blocking antibody against mouse TLR2 or TLR4/MD2 (20 µg/mL) (InvivoGen, San Diego, CA) or with an isotype control antibody (InvivoGen). The purified polysaccharide from the melon fly pupae (1 ng/mL) or LPS (1 ng/mL) was then added to the RAW264 macrophages and the cells were further incubated for 20 h at 37°C in 5% CO_2_. The levels of NO in the culture medium of RAW264 cells were measured using the Griess reagent system kit as described above.

### Western blot analysis

Cells (1×10^6^) were washed with PBS, resuspended in PBS containing 1% Triton X-100 and one tablet of protease inhibitor cocktail (Roche, Mannheim, Germany), and sonicated once for 30 sec. The lysate was then centrifuged at 20,400×g for 30 min at 4°C to remove insoluble material. For nuclear protein extraction, the cells were washed three times with ice-cold PBS, collected with a cell scraper, and harvested by centrifugation. The nuclear proteins were prepared using a NE-PER Nuclear and Cytoplasmic Extraction Reagents (Thermo Scientific, Rockford, IL) as described by the manufacturer’s protocol. Aliquots of 10–20 µg of denatured protein were separated by 7.5% or 12.5% SDS-PAGE and transferred to PVDF membranes (Millipore, Billerica, MA) by electroblotting. The primary antibodies against mouse iNOS and NF-κB p65 (rabbit polyclonal antibody; Santa Cruz Biotechnology, Santa Cruz, CA), and I-κBα (mouse monoclonal antibody; Cell Signaling Technology, Danvers, MA) were diluted 1∶5,000 in immunoreaction enhancer solution (Can Get Signal Solution 1; Toyobo, Osaka, Japan). For total protein level normalization, α-tubulin (mouse monoclonal antibody; Sigma Aldrich, St Louis, MO) was used as a loading control. For normalization of nuclear protein levels, lamin A/C (goat polyclonal antibody; Santa Cruz Biotechnology) was used as a loading control. The membrane was incubated at 4°C overnight and the secondary antibody, either anti-rabbit, anti-mouse or anti-goat IgG alkaline phosphatase (GE Healthcare), diluted 1∶20,000 in immunoreaction enhancer solution (Can Get Signal Solution 2; Toyobo) was added. The membrane was the incubated for 1 h at room temperature. After washing, the alkaline phosphatase signal was detected using CDP-*sta*r Detection Reagent (GE Healthcare) and observed using an ImageQuant LAS 4000 (Fujifilm, Tokyo, Japan).

### Electrophoresis

Sodium dodecyl sulfate-polyacrylamide gel electrophoresis (SDS-PAGE) was performed using a 5%–20% precast e-PAGEL gel (ATTO, Tokyo, Japan). After electrophoresis, the gel was stained with Coomassie Brilliant Blue R-250. The molecular weights of the protein bands in the gel were estimated using molecular mass markers (Bio-Rad Laboratories, Hercules, CA).

### Isolation of total RNA and Real-time quantitative RT-PCR

Total RNA was isolated from RAW264 cells using RNeasy Plus Mini kit (Qiagen, Hilden, Germany) according to the manufacturer’s protocol. For the real-time PCR assay, 500 ng total RNA was reverse-transcribed using the Qiagen QuantiTect Reverse Transcription kit. Real-time PCR analysis was performed on a Bio-Rad CFX96 Real-time PCR Detection system (Bio-Rad Laboratories) using SsoFast EvaGreen Supermix (Bio-Rad Laboratories). The gene specific primers used in this study were same as those reported by Xia *et al.* [25]. The thermocycling conditions comprised 1 min at 95°C and then 50 cycles with 2 s at 95°C, 10 s at 60°C. The comparative CT method was used to determine the amount of target, normalized to an endogenous reference (GAPDH) and relative to a calibrator (2^−ΔΔCt^) using the CFX Manager 2.0 software (Bio-Rad Laboratories). All RT-PCR experiments were performed in triplicate.

### Statistical analysis

All results presented in this study are expressed as the mean ± SEM. All values were analyzed by a one-way analysis of variance followed by a Bonferroni multicomparison test using KaleidaGraph statistical software. Significance was set at *p*<0.05.

## Results

### *In vitro* effects of melon fly pupal extract on NO production by RAW264 macrophages

**Figure 1:**
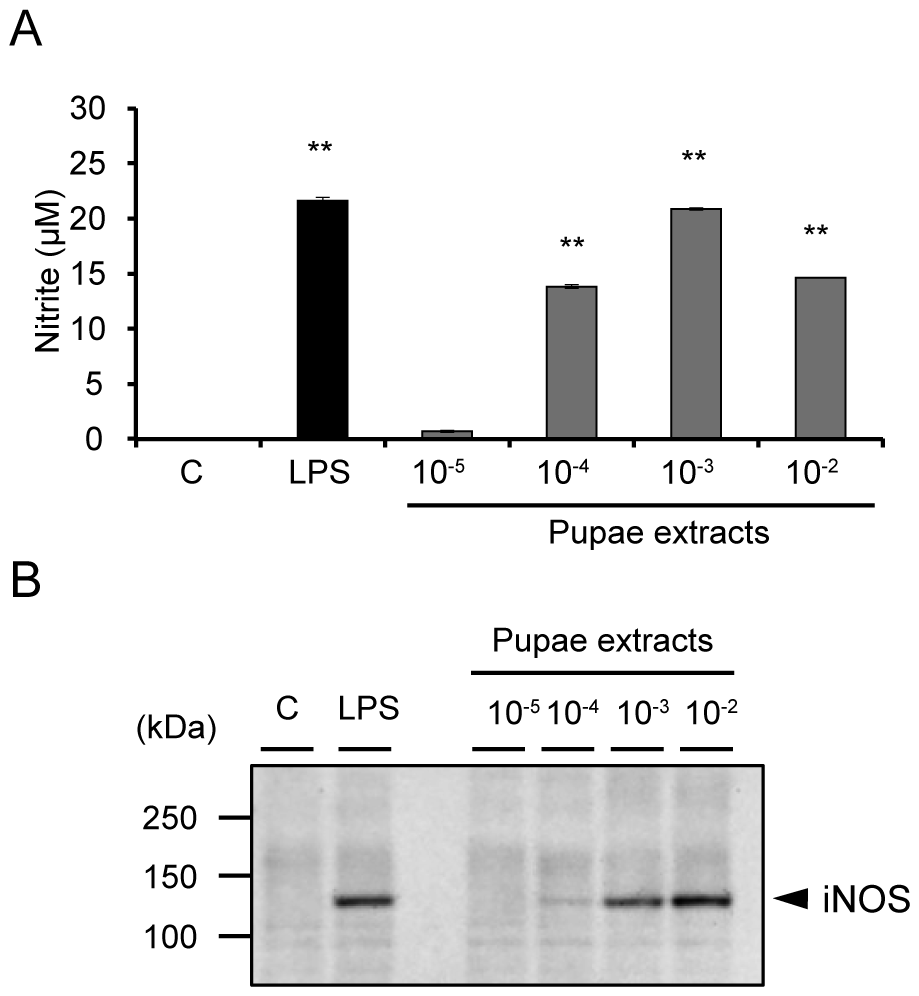
Melon fly pupal extract stimulates NO production in RAW264 cells. (A) RAW264 cells were incubated with various concentrations of melon fly pupal extract for 20 h. The nitrite levels in the culture medium were then measured using the Griess assay, as described in the Materials and Methods. (B) Expression of iNOS protein in the melon fly pupal extract-treated cells determined by western blot analysis using an antibody specific for murine iNOS. Cells were incubated with varying concentrations of the melon fly pupal extract for 6 h. Results are given as means ± SEM. **p<0.01 versus control.

Initiation of the innate immune response is mediated by PRRs such as TLRs that recognize various pathogen components. Upon pathogen recognition, signal transduction pathways are initiated that lead to the induction of various cytokines and/or production of NO via iNOS. Previous studies have reported that TLRs recognize a wide variety of molecules, including polysaccharides. To investigate whether melon fly pupae contains substances that activate the innate immune system, we analyzed NO production, a major mediator of immunostimulatory activity, in RAW264 cells treated with crude extract from melon fly pupae. The pupal extract induced NO production in RAW264 cells in a dose-dependent manner (Fig. 1A). In macrophages such as RAW264 cells, NO is synthesized by iNOS protein. To investigate whether the expression of iNOS protein is induced by stimulation with pupal extract, the expression level of iNOS protein was monitored by western blot analysis (Fig. 1B). Stimulation with pupal extract induced a dose-dependent increase in the level of iNOS protein.

### Analysis of the characteristics of the innate immune activator in pupal extract

**Figure 2:**
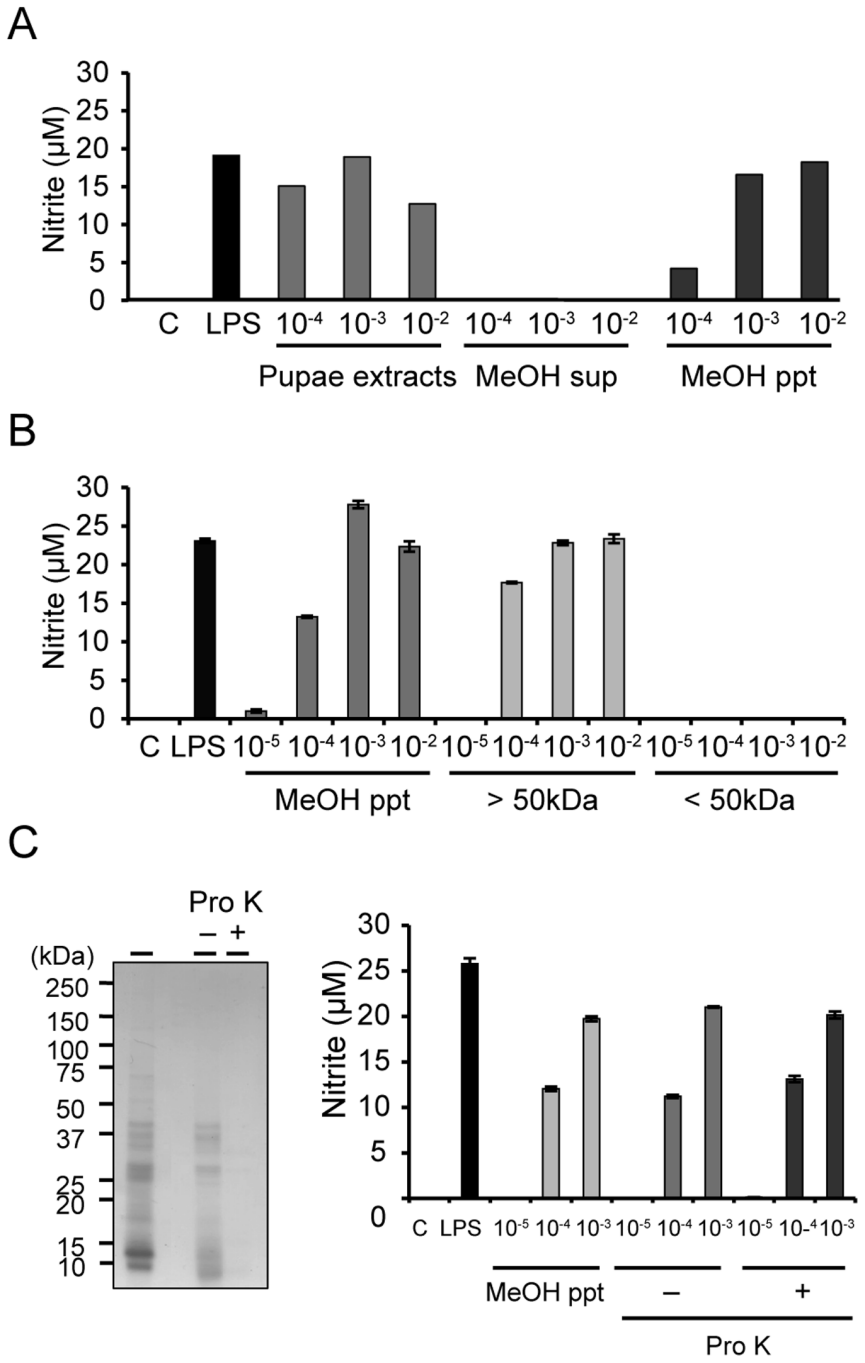
Characterization of the innate immune activator in melon fly pupal extract. (A) RAW264 cells were stimulated with a methanol extract or precipitate of melon fly pupal extract for 20 h and the nitrite levels in the culture medium were measured using the Griess assay. (B) RAW264 cells were stimulated with the methanol precipitate of melon fly pupal extract treated with ultrafiltration for 20 h and the nitrite levels in the culture medium were measured by Griess assay. (C) SDS-PAGE analysis of the methanol precipitate fraction of melon fly pupal extract, treated or untreated with proteinase K (1.5 U/mL at 55°C overnight). RAW264 cells were stimulated with the untreated or proteinase K-treated methanol precipitate and nitrite levels in the culture medium were then measured by Griess assay.

PRRs such as TLRs recognize a wide variety of pathogen-associated molecules, including proteins, polysaccharides, and nucleic acids, and subsequently activate the innate immune response, including NO production. To investigate the characteristics of insect substances that would activate mammalian innate immunity, we performed methanol extraction of immune activators from melon fly pupae (Fig. 2A). *In vitro* analysis revealed that the methanol precipitate of these pupal extracts induced NO production in RAW264 cells. To investigate the molecular size of the putative innate immune activator, we used ultrafiltration with YM-50 membranes to separate substances from the methanol precipitate (Fig. 2B). *In vitro* analysis subsequently showed that the molecular size of the immune activator was larger than 50,000. We thus expected that immune activators from the melon fly pupae would be high-molecular-weight compounds such as proteins, polysaccharides, or nucleic acids.

To investigate whether the insect immune activators were proteins, we assessed in RAW264 cells the NO-producing activity of the methanol precipitate when treated with proteinase K (Fig. 2C). SDS-PAGE analysis indicated that various proteins in the methanol precipitate had been degraded by proteinase K treatment but this had no effect on the NO-producing activity of the precipitate *in vitro*.

### Purification of the innate immune activator from pupal extract

**Figure 3:**
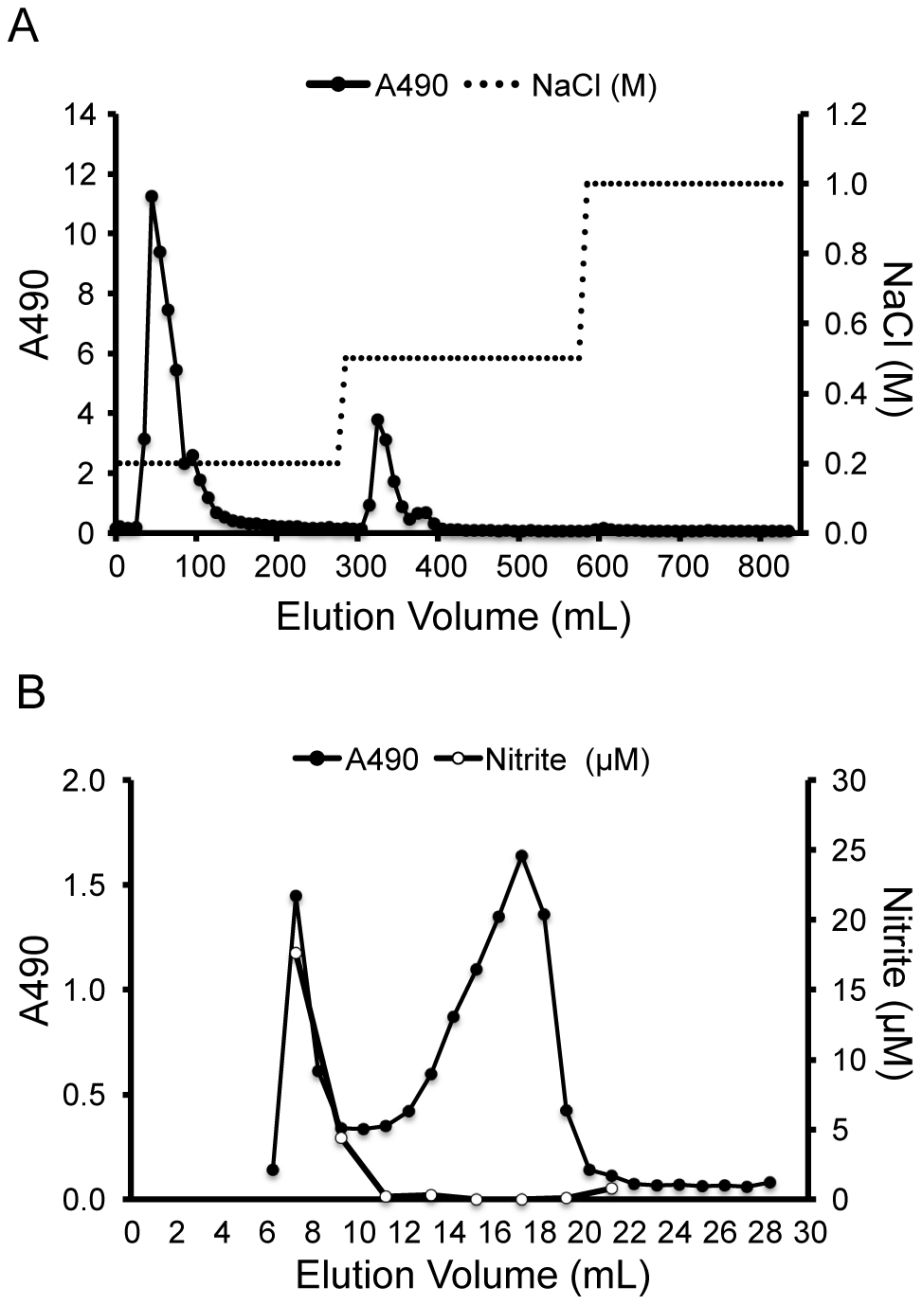
Chromatography profiles of the polysaccharides contained in melon fly pupal extract. (A) Anion-exchange chromatography profile of the polysaccharide fractions of melon fly pupal extract on a DEAE-cellulose column eluted with sequential NaCl aqueous solutions (0.2, 0.5, and 1.0 M). (B) Gel filtration chromatography profile of acidic polysaccharide fractions of melon fly pupal extract. Polysaccharides were passed through a Superpose 6 column. Fractions were collected and the sugar content was monitored using the phenol-H_2_SO_4_ method (filled circle, OD490 nm). RAW264 cells were incubated with these diluted fractions for 20 h and the nitrite levels in the culture medium were measured by Griess assay (Open circle, OD540nm).

Based on our proteinase K experiment results, we speculated that the innate immune activator from the melon fly pupae was likely to be a glycoconjugate, such as a polysaccharide or peptidoglycan. Pupal extracts were added to four volumes of 100% (v/v) methanol to remove soluble materials such as free sugars and amino acids, and to precipitate the glycoconjugates. The dried methanol precipitate was then dissolved in 20 mM Tris-HCl (pH8.0) overnight for 4°C. The resulting supernatant fraction obtained by centrifugation was collected and purified by anion-exchange chromatography on a DEAE-cellulose column following elution with sequential NaCl solutions (0.2, 0.5, and 1.0 M NaCl) and detected by a phenol-sulfuric acid assay (Fig. 3A). Two fractions containing sugars (eluted with 0.2 and 0.5 M NaCl) were collected and both were found to induce NO production *in vitro*. The 0.2 M NaCl-eluted fraction had strong immunostimulatory activity and was purified by gel filtration chromatography on a Superose 6 column; two fractions containing sugars (the high- and low-molecular-weight polysaccharides) were thereby collected (Fig. 3B). *In vitro* analysis subsequently showed that only the high-molecular-weight polysaccharide activated the innate immune system. We named this bioactive Diptera polysaccharide “dipterose”.

### Structural characteristics

**Figure 4:**
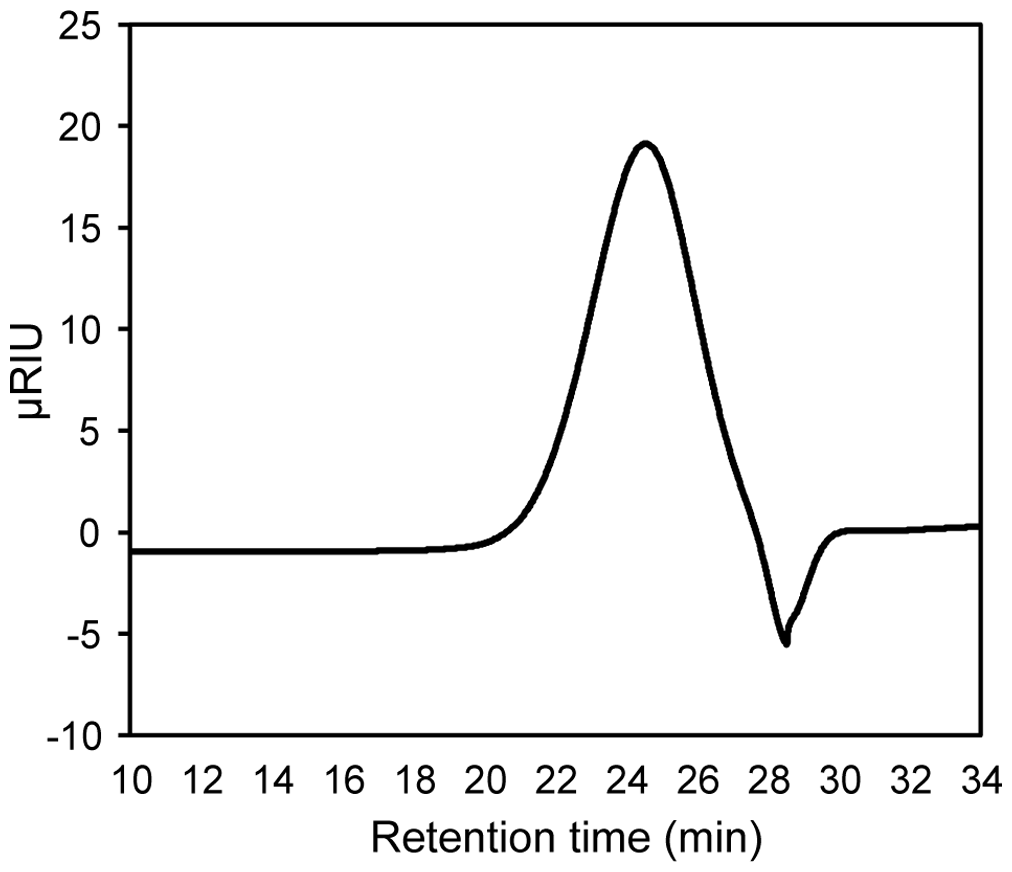
HPLC profiles of dipterose on a Shodex SB-807 HQ column. The results indicate that dipterose comprises homogeneous polysaccharides.

To investigate whether dipterose was homogeneous, we performed high-performance size-exclusion chromatography (HPSEC) on a Shodex SB-807 HQ column (Fig. 4). Dipterose was detected as a single symmetrical peak, indicating a single homogeneous polysaccharide. Moreover, the average molecular weight of dipterose was estimated at 1.01×10^6^ via analysis using HPSEC and with reference to pullulan P-series standard samples of known molecular weights.

### Identification of the monosaccharide composition of dipterose

**Figure 5:**
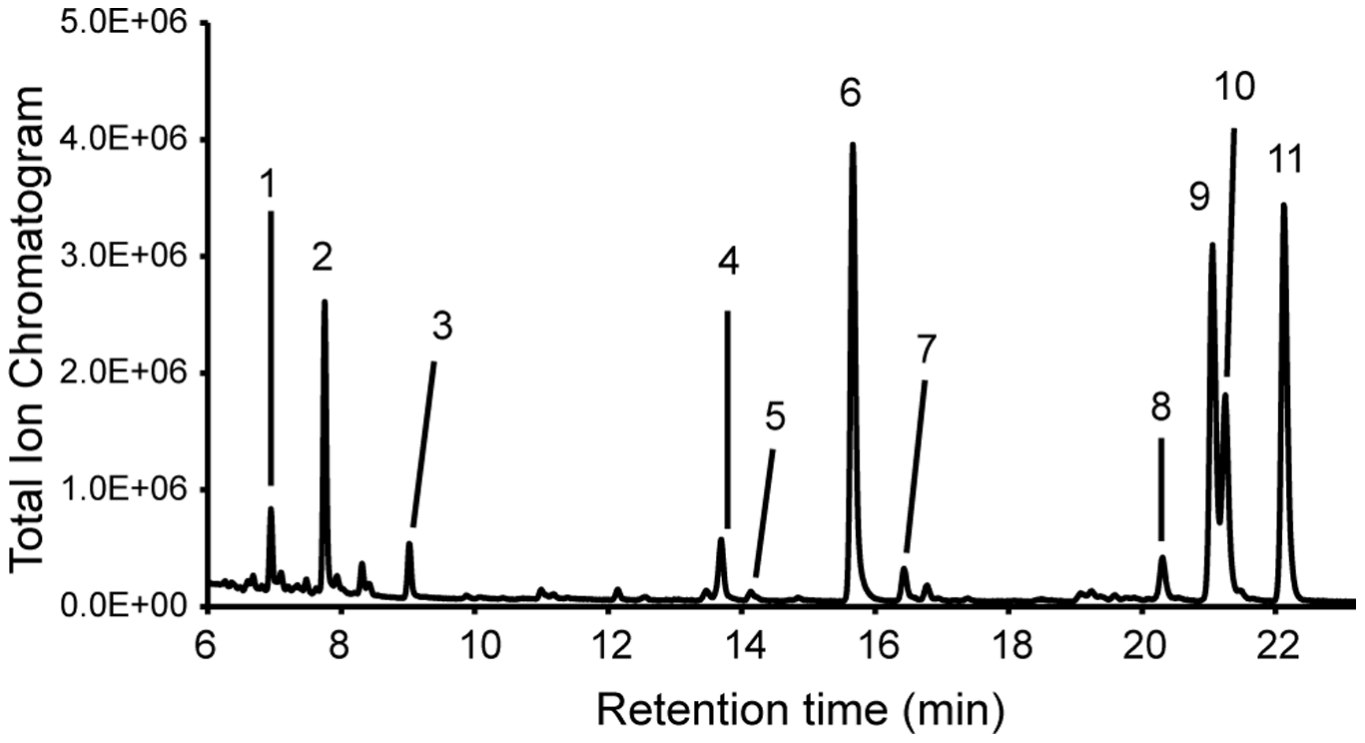
GC-MS chromatogram of monosaccharides of dipterose. Peaks: 1, N-acetyl-D-galactosamine; 2 and 3, N-acetyl-D-glucosamine; 4, myo-inositol (internal standard); 5, D-ribose; 6, L-rhamnose; 7, L-fucose; 8, D-glucuronic acid; 9, D-glucose; 10, D-mannose; 11, D-galactose.

The monosaccharide composition of dipterose was determined by GC-MS analysis of trimethylsilylated diethyl dithioacetate derivatives (Fig. 5). This analysis showed that dipterose comprises nine monosaccharides–D-glucose, D-mannose, D-galactose, D-ribose, L-rhamnose, L-fucose, D-glucuronic acid, N-acetyl-D-glucosamine, and N-acetyl-D-galactosamine–at a molar ratio of 18.2∶8.0∶16.6∶0.3∶21.2∶1.4∶6.2∶19.6∶8.5, respectively (Table 1).

**Table 1:** Monosaccharide composition and molar ratio in dipterose.

Name	Glc	Man	Gal	Rib	Rha	Fuc	GlcA	GlcNAc	GalNAc
Molar ratio	18.2	8.0	16.6	0.3	21.2	1.4	6.2	19.6	8.5

### Changes in dipterose activity according to the developmental stage of the melon fly

**Figure 6:**
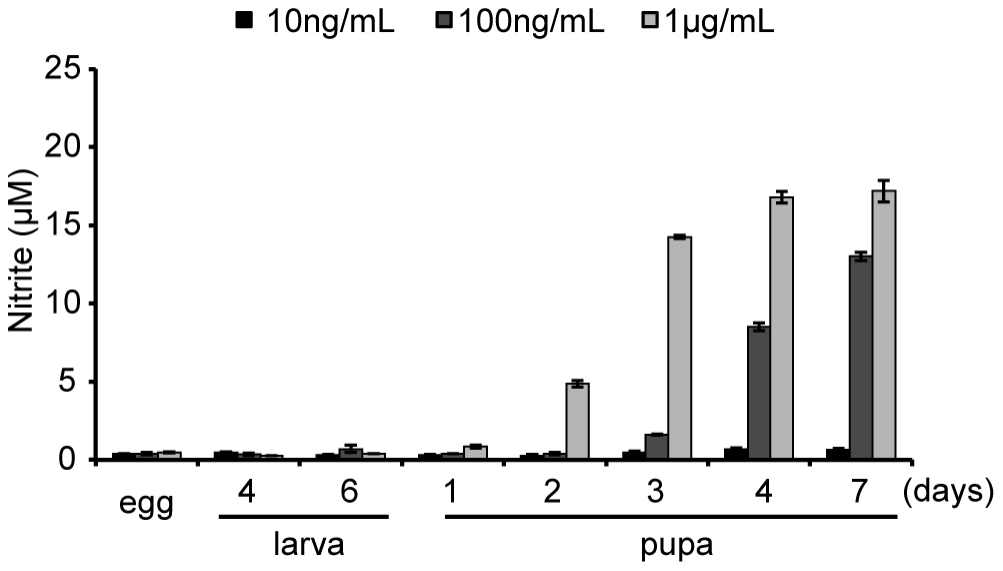
Sequential changes in dipterose activity with the developmental stage of the melon fly. Melon fly eggs, larvae, or pupae were homogenized with four volumes of PBS. The homogenates were then centrifuged at 20,400×g for 30 min at 4°C and the sugar content of the supernatants were measured using the phenol-H_2_SO_4_ method. RAW264 cells were incubated with these diluted supernatants for 20 h and the nitrite levels in the culture medium were measured by Griess assay.

To evaluate changes in dipterose activity over the developmental stage of the melon fly, we measured the NO-producing activities of eggs, two larval stages, and five pupal stages in RAW264 cells *in vitro* (Fig. 6). NO production was detected from the pupal stage and increased as the developmental stage progressed.

### Effects of dipterose on the activation of the innate immune system *in vitro*

**Figure 7:**
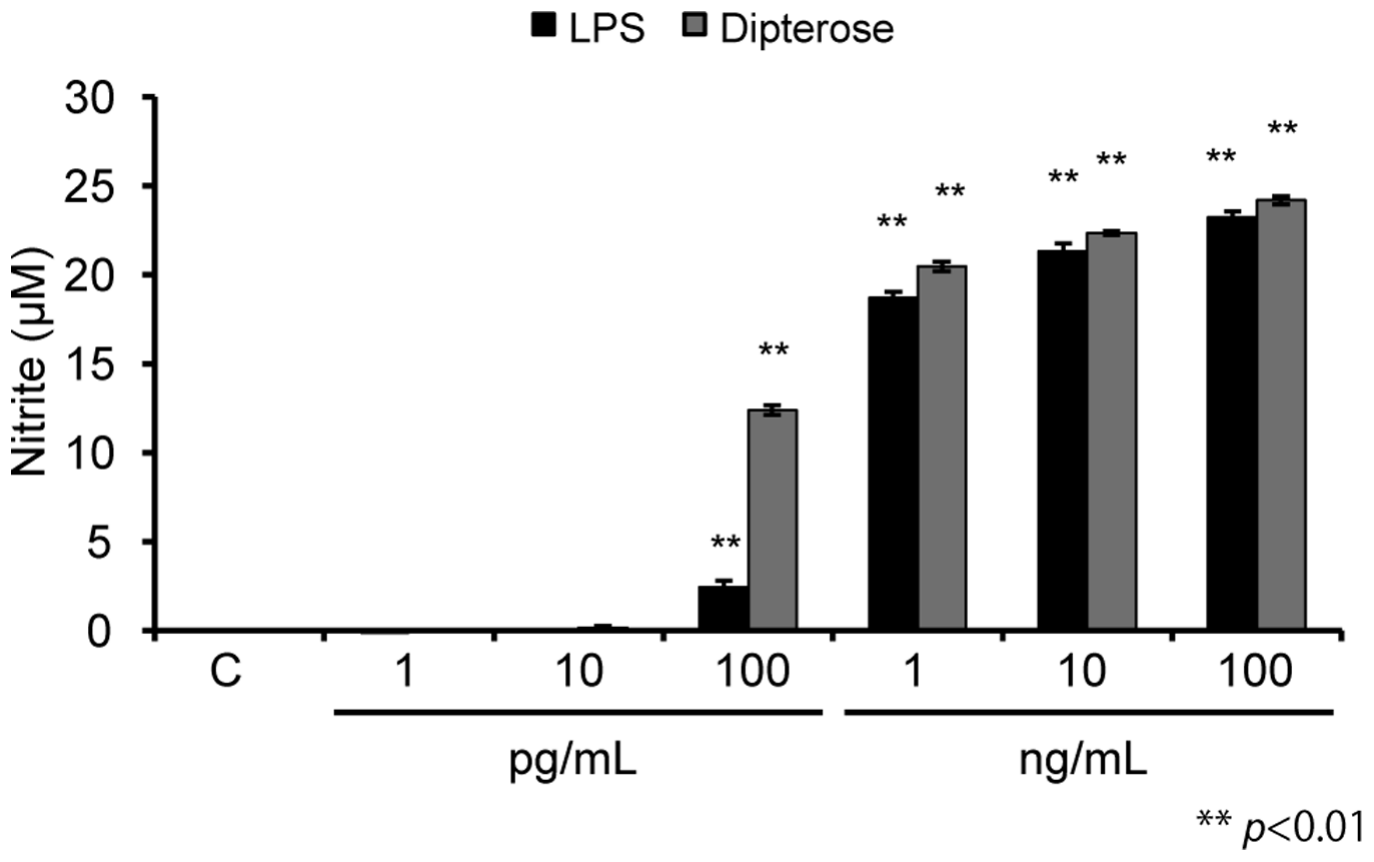
Dipterose stimulation induces NO production in macrophages. RAW264 cells were stimulated with various concentrations of dipterose or LPS for 20 h and nitrite concentrations in the culture medium were measured as described in the Materials and Methods. Results are given as means ± SEM. **p<0.01 versus control.

To evaluate the immunostimulatory activity of dipterose, we analyzed NO production in RAW264 cells treated with various concentrations of this polysaccharide. An *in vitro* assay indicated that the addition of 100 pg/mL dipterose induced NO production in RAW264 cells; the immunostimulatory activity of dipterose thus approximated that of LPS, a known potent immunostimulator (Fig. 7).

### Effect of TLR2 and TLR4 inhibition on NO-producing activity of dipterose *in vitro*

**Figure 8:**
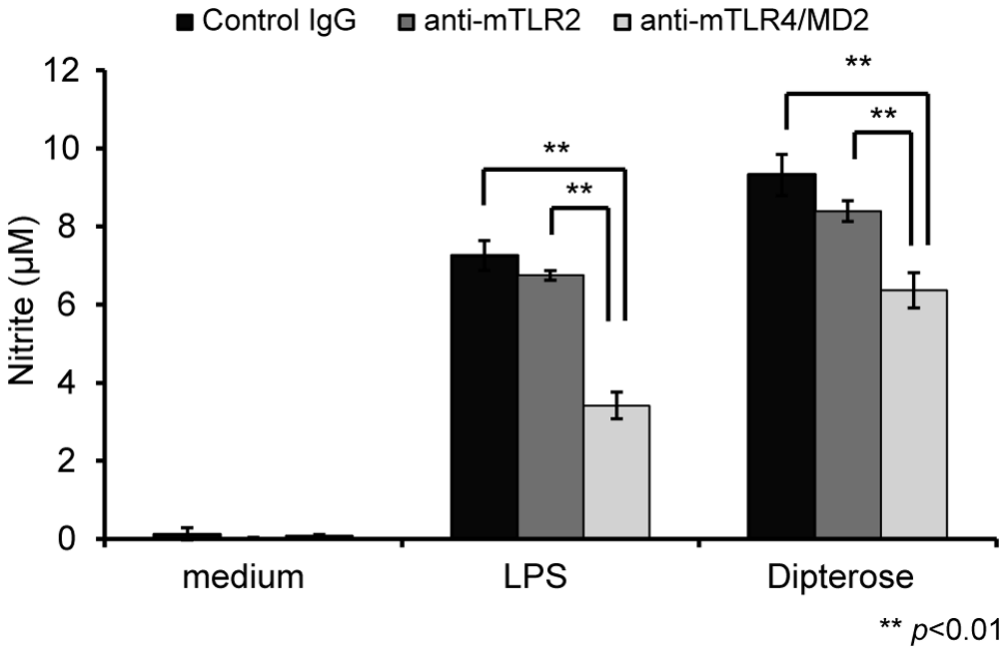
Dipterose induces NO production in macrophages through TLR4. Macrophages were incubated with neutralizing antibody to TLR2 and TLR4 (20 µg/mL) or isotype control IgG for 30 min, followed by 20 h of incubation with dipterose. NO production in the medium was then measured using Griess reagent solution. Results are given as means ± SEM. **p<0.01 versus control.

Previous studies have reported that TLR2 and TLR4 activate the innate immune response by recognizing glycans such as zymosan and LPS, cell wall components of yeast and gram-negative bacteria, respectively, and play an important role as a bridge between innate and adaptive immunity [14]. To investigate whether dipterose induces NO production in RAW264 cells via TLR2 or TLR4, we used antibodies against these receptors to inhibit their function and thereby determine their effect on NO production by RAW264 cells (Fig. 8). Anti-mTLR4/MD2 antibodies significantly inhibited dipterose-stimulated NO production, whereas anti-mTLR2 antibodies failed to do so. These data suggest that dipterose principally stimulates the innate immune system through the activation of TLR4.

### Effect of dipterose on the expression of cytokines and IRF3 and IRF7 mRNA

**Figure 9:**
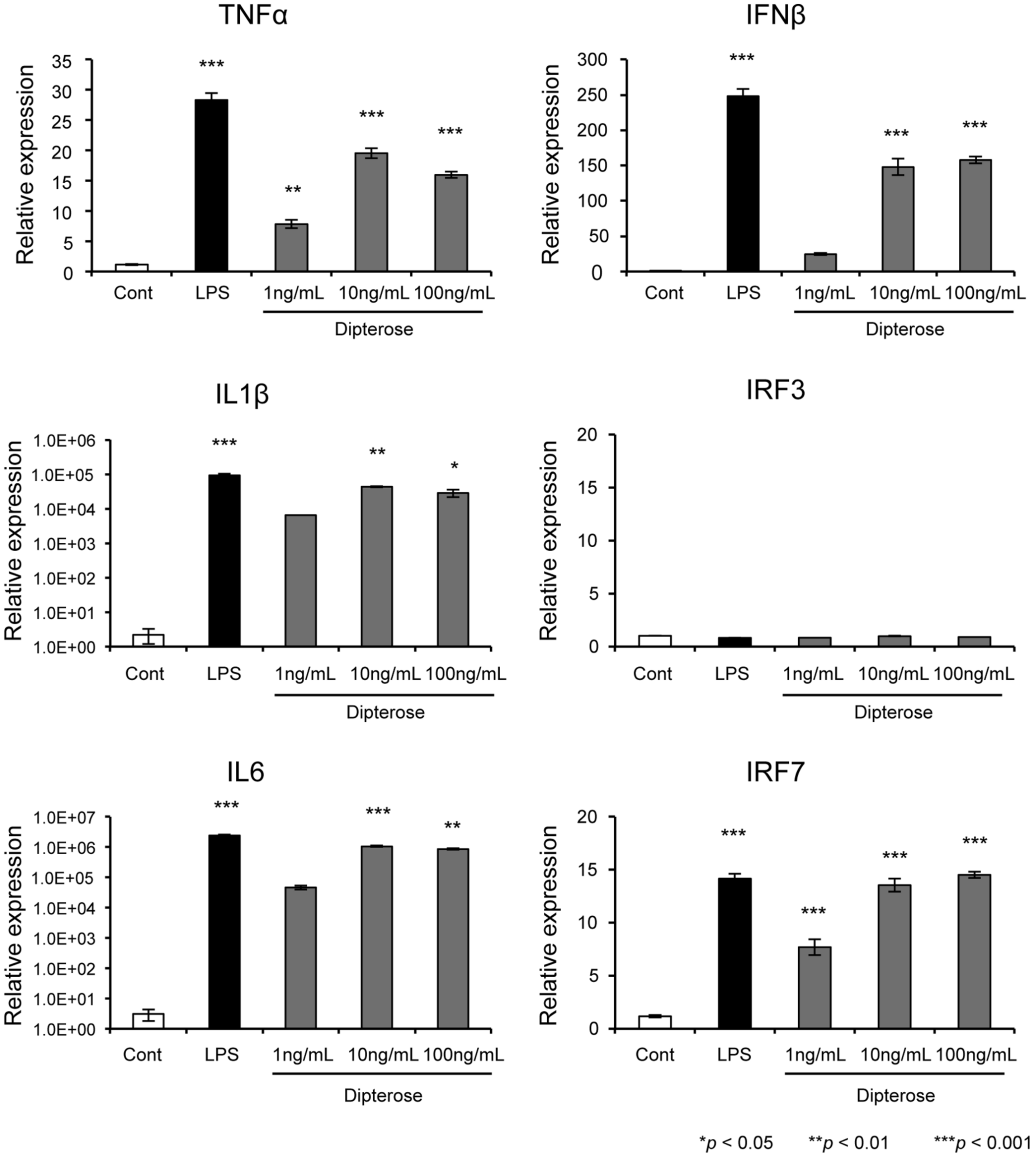
Dipterose induces the expression of cytokines and IRF7 via TLR4 signaling transduction pathway. RAW264 cells were stimulated with various concentrations of dipterose or LPS. The mRNA expression of the indicated cytokines and of IRF3 and IRF7 was measured using real-time PCR at 6 h after dipterose or LPS treatment. The results represent the means ± SEM of at three samples for each gene analyzed. *p<0.05, **p<0.01 and ***p<0.001 as compared with control group.

Previous studies have reported that TLR4-mediated signaling is transduced by two distinct MyD88-dependent and MyD88-independent pathways and induces the expression of various genes [13], [14]. Activation of the MyD88-dependent pathway via TLR4 induces the expression of proinflammatory cytokines such as TNFα, IL1β, and IL6. On the other hand, the MyD88-independent pathway induces the expression of IFNβ, IRF7 and IFN-inducible genes through IRF3 activation [13], [25]. To determine whether dipterose induces cytokine expression in RAW264 cells, we analyzed the expression of dipterose-induced cytokine, IRF3 and IRF7 by RAW264 cells (Fig. 9). The expression of several proinflammatory cytokines, such as TNFα, IL1β, IL6, and IFNβ was significantly increased at 6 hours after dipterose treatment in a dose-dependent manner. Dipterose also significantly induced the expression of IRF7 mRNA, but not IRF3 mRNA, at 6 hours post-treatment. Significantly, these gene expression profiles associated with dipterose correspond to those of LPS, a known TLR4 ligand.

### Effect of dipterose on TLR4 signaling

**Figure 10:**
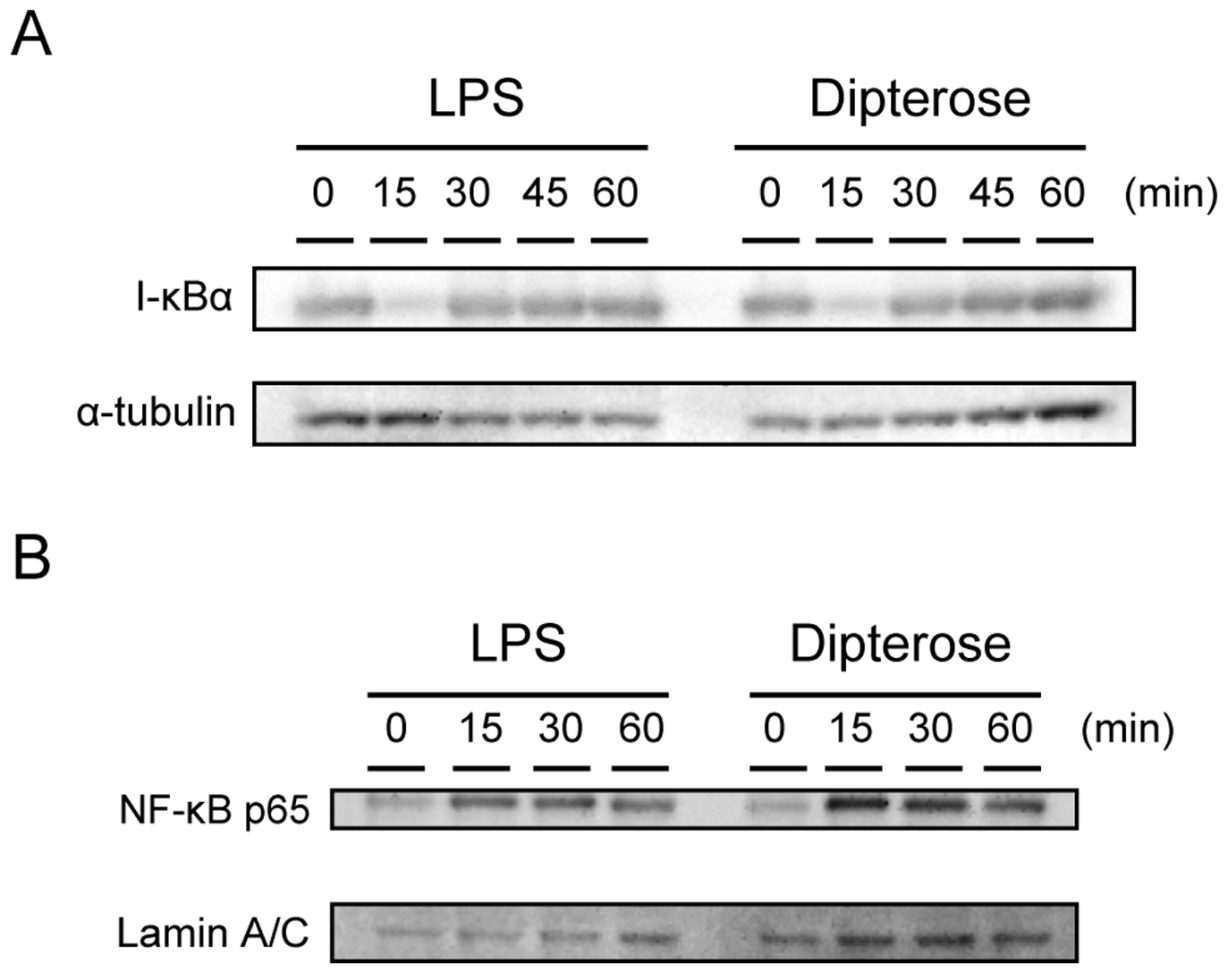
Dipterose induces the activation of NF-κB in macrophages. (A) RAW264 cells were stimulated with dipterose or LPS for 0, 15, 30, 45 or 60 min. I-κBα was detected by western blot analysis. α-tubulin is shown as a protein loading control. (B) RAW264 cells were treated with dipterose or LPS for 0, 15, 30 or 60 min. NF-κB p65 translocation into the nucleus was measured by western blot analysis. Lamin A/C is shown as a protein loading control.

Dipterose induces cytokine gene expression through TLR4 activation in RAW264 cells. NF-κB is an important translational factor functioning downstream of the TLR4 signal transduction pathway. To investigate whether dipterose activities are mediated via NF-κB, we analyzed the degradation of the NF-κB inhibitory protein, I-κBα, by immunoblotting and also assessed the translocation of NF-κB p65 to the nucleus upon exposure to dipterose. The treatment of RAW264 cells with dipterose induced a rapid degradation of I-κBα in a comparable manner to LPS treatment (Fig. 10A). Reduced I-κBα protein levels were observed 15 min after dipterose treatment but recovered to basal levels after 60 min. Furthermore, the translocation of NF-κB p65 to the nucleus was found to be increased at 15 min post-treatment in dipterose-induced RAW264 cells (Fig. 10B), which is in correlation with the result of I-κBα degradation.

## Discussion

Insects are currently receiving considerable worldwide attention not only as sources of food and feed, but also as living organisms capable of producing a variety of useful substances [1]. Until now, functional polysaccharides from insects have gone largely unnoticed, although various useful substances, such as low-molecular-weight compounds and peptides, have been identified [4], [7], [8], [12]. In our current study, we describe the identification and characterization of dipterose, a novel polysaccharide isolated from melon fly pupae. The melon fly is a significant worldwide pest that attacks fruit and vegetable crops. Our data clearly demonstrate that dipterose stimulates TLR4 and activates the innate immune system in a macrophage RAW264 cell line.

Macrophages, along with other innate immune cells, play an important role in the host defense system against a variety of pathogenic infections. Furthermore, the activation of innate immune cells such as macrophages is also important for triggering acquired immunity. The recognition of pathogen components by macrophages leads to the production of cytokines and NO, a cytotoxic molecule, which are essential for effective pathogen elimination [14], [26], [27]. Hence, NO production by macrophages is widely used as a marker of activation of the innate immune response [28]. In our current study, we describe our identification of a water-soluble immune activator contained in extracts of melon fly pupae. We used NO production in mouse macrophage RAW264 cells as an indicator of immunostimulation to screen for this compound. Although chitin and chitin derivatives of the insect exoskeleton have been reported to activate the immune response in mammals [22], the immune activator contained in melon fly pupae is different due to its water solubility. Hence, the immune activator contained in melon fly pupae appeared to be a novel natural product and not chitin or a chitin derivative.

NO production by macrophages is induced by a wide variety of substances, such as peptides, oligodeoxynucleotides, and glycoconjugates [28], [29]. Although insects have various bioactive compounds and peptides, analysis by ultrafiltration and proteinase treatment had suggested previously that the immune activator from pupae was a glycoconjugate such as a polysaccharide. Various natural glycoconjugates such as polysaccharides derived from plants, fungi, and bacteria that form cell walls stimulate NO production by macrophages [28]. To our knowledge however, there has been no previous report that glycoconjugates are produced in animals, including insects. In our current study, we identified an acidic polysaccharide in pupal extracts with a molecular weight of 1.01×10^6^ that stimulated NO production by macrophages. Moreover, GC-MS analysis revealed that this bioactive molecule is a novel polysaccharide composed of nine monosaccharides which we termed “dipterose”.

Although dipterose is predominantly composed of L-rhamnose, insects, like other animals and in contrast to plants, fungi, and bacteria, are unable to synthesize NDP-rhamnose, an activated monosaccharide [30]. NDP-sugars, including UDP- and dTDP-rhamnose, are synthesized through *de novo* and salvage pathways and serve as substrates in the synthesis of glycan [31]. In plants, UDP-rhamnose is required for primary cell wall polysaccharides and various L-rhamnose–containing natural organic compounds such as flavonoids, terpenoids, and saponins [32]–[34] and is synthesized through a *de novo* pathway from UDP-D-glucose [35], [36]. Although a salvage pathway for UDP-rhamnose remains to be identified in plants, there is evidence for such a pathway because UDP-glucose pyrophosphorylase catalyzes the formation of various UDP-sugars from monosaccaharide-1-phospates at the end of the salvage pathway [31], [37]. Melon fly is a phytophagous insect whose larvae feed on the pulp of gourds, fruits vegetables and fruits such as papaya and mango [38]. Hence, it was feasible that a unique salvage pathway was active in this insect. On the other hand, we found from our current analyses that the dipterose levels increased in the pupal stages even though pupae do not feed. This result suggests that the melon fly synthesizes UDP-rhamnose from other UDP-sugars through a *de novo* pathway and then uses these products as substrates for the synthesis of dipterose.

Previous studies have reported that a number of insects have bacterial endosymbionts that can have a mutualistic relationship with their hosts, providing them with nutrients such as amino acids and vitamins, or that involves intracellularly parasitizing and negatively affecting them [39]–[41]. However, there are no reports of bacterial endosymbionts that have achieved a mutualistic relationship with the melon fly, which feeds mainly on cucurbitaceous plants but not on plant sap or blood [42], [43]. Moreover, there is no melon fly which infected with reproductive manipulators such as *Wolbachia* to be able to mass-produce sterile insects throughout the year. These results suggest that a novel polysaccharide composed of a variety of sugars including L-rhamnose is synthesized by the melon fly itself without the effect of bacterial endosymbionts.

Plants and fungi are now known to have various bioactive polysaccharides that induce cytokine and NO production by macrophages [28], [44]. The cell walls of plants and fungi predominantly contain various polysaccharides comprising species-specific monosaccharides. Although previous studies have reported that high-dose treatments of macrophages with many of these polysaccharides activate the innate immune response, we show from our current data that a very low concentration of dipterose can do this at a similar potency to LPS, an immunostimulator and major component of the cell membrane of gram-negative bacteria. The polysaccharide structure is an important determinant of the activation of innate immune cells such as macrophages. Our current findings suggest that dipterose has a characteristic structure that is a potent stimulator of mammalian macrophages.

Activation of the innate immune response by polysaccharides is triggered by their recognition by PRRs such as TLRs. Although TLRs recognize structures that are conserved among various pathogens [45], TLR2 and TLR4 have been well characterized as sensors that recognize ligands containing carbohydrate moieties such as peptidoglycans, LPS, and natural polysaccharides [45], [46]. We show from our current analyses that the inhibition of TLR4, but not TLR2, leads to the suppression of the NO production-stimulating activity of dipterose. The NO production induced by dipterose was also partially inhibited by anti-TLR4 antibody treatment. Four different classes of PRR families have been identified to date [45]. Therefore, dipterose may induce the innate immune response via other PRR pathways in addition to TLR4.

Stimulation of TLR4 facilitates activation of MyD88-dependent and -independent pathways leading to the production of proinflammatory cytokines and IFNβ and the expression of IFN-inducible genes [13], [25]. In addition, the activation of innate immunity by TLR stimulation is important for the activation of acquired immunity [14], [45]. The expression profiles of dipterose-induced cytokines indicate that this polysaccharide induces proinflammatory cytokines such as TNFα, IL1β, IL6, and IFNβ through the activation of MyD88-dependent and MyD88-independent signaling. Furthermore, the stimulation of dipterose promoted the degradation of I-κBα and the translocation of NF-κB to the nucleus. These results further indicate that dipterose activates the innate immune system via TLR4 signaling.

Toll receptors in insects and TLRs in mammals are critically important for innate immunity, suggesting that these receptors are evolutionarily conserved from insects to mammals [47], [48]. In insects, a number of genes encoding a Toll-related receptor have been identified [49]–[51]. Some of these receptors function as cytokine or pattern recognition receptors and have been shown to be involved in the innate immune response [52], [53]. However, there is little information available on the functions of other Toll-related receptors and their ligands. In the silkworm, the expression of Toll-related receptors changes during development and increases for some of these receptors at the pupal and adult stages [50]. Hence, as dipterose is synthesized in the pupal stage, it may be an endogenous ligands of a Toll-related receptor that activates the innate immune system itself.

In conclusion, we have identified a novel acidic polysaccharide from melon fly pupae termed dipterose that activates the innate immune system of mammalian macrophages. Surprisingly, we found that dipterose is predominately composed of L-rhamnose, despite the fact that insects and higher animals lack the ability to synthesize this compound. This suggests that insects have unique L-rhamnose biosynthesis pathways. Furthermore, we demonstrate from our current analyses that dipterose activates the innate immune system via TLR4, inducing the production of proinflammatory cytokines and IFN-β and the expression of IFN-inducible genes. Our findings indicate that dipterose activates the innate immune system in macrophages and may enhance the immune response to various pathogens.
